# A168 DIFFERENTIAL ASSOCIATION OF DIETARY FIBER INTAKE WITH METABOLOMIC AND PROTEOMIC SIGNATURES LINKED TO THE RISK OF CD

**DOI:** 10.1093/jcag/gwae059.168

**Published:** 2025-02-10

**Authors:** J Kim, C McShane, M Xue, S Lee, H Leibovitzh, J Shao, R Khorasaniha, A Griffiths, H Armstrong, P Moayyedi, K Croitoru, W Turpin

**Affiliations:** Toronto, ON, Canada; Toronto, ON, Canada; Toronto, ON, Canada; Toronto, ON, Canada; Toronto, ON, Canada; Toronto, ON, Canada; University of Manitoba, Winnipeg, MB, Canada; SickKids, Toronto, ON, Canada; University of Manitoba, Winnipeg, MB, Canada; McMaster University, Hamilton, ON, Canada; Toronto, ON, Canada; Toronto, ON, Canada

## Abstract

**Background:**

Epidemiologic studies have shown that increased dietary fiber intake may be associated with a reduced risk of developing Crohn’s disease (CD). Our previous research identified specific patterns of serum proteomics and metabolomics associated with CD onset.

**Aims:**

We aimed to understand how specific dietary fibers might be associated with risk biomarkers of CD in a high-risk population.

**Methods:**

In the CCC-GEM Project, a nested case-control cohort was established involving healthy first-degree relatives (FDRs) of individuals with CD. This cohort was matched in a 1:4 ratio, comparing those who developed CD with those who remained healthy. The intake of five energy-adjusted dietary fibers, including inulin, fructooligosaccharides (FOS), β-glucan, pectin, and arabinoxylan, was assessed using a food frequency questionnaire completed at recruitment. Of the serum markers measured at recruitment, 63 metabolites (Metabolon®) and 25 proteins (Olink®), and C-reactive protein (Prometheus®) were associated with future CD risk; 34 metabolites and 15 proteins were associated with increased CD risk, while 29 metabolites and 10 proteins were protective against CD risk. To evaluate the association between dietary fibers and these serum markers, we used generalized estimating equations, considering a false discovery rate-adjusted p < 0.05 as significant.

**Results:**

Among 215 healthy FDRs, 41 developed CD during a median follow-up of 9.4 years, while 173 remained healthy. We found that 14 metabolites and eight proteins previously associated with an increased risk of developing CD in future were negatively associated with the intake of at least one dietary fiber, whereas six metabolites and five proteins linked to a decreased risk of CD development were positively associated with dietary fibers. In particular, the intake of inulin, FOS, and β-glucan was negatively associated with the CD-associated markers TREM1, MMP9, HGF, and sphingomyelin species (p < 0.001), and positively associated with isocitrate (p < 0.001), previously linked to a reduced CD risk. Furthermore, the consumption of inulin, FOS, β-glucan, anarabinoxylan were negatively associated with C-reactive protein levels (p < 0.05).

**Conclusions:**

Our findings suggest that select fermentable dietary fiber intake may be protective against the development of CD by influencing the abundance of specific proteins and metabolites associated with CD risk. Further research is needed to directly explore the mechanisms of specific fermentable dietary fibers on CD development.

Submitted on behalf of the Crohn’s and Colitis Canada GEM Project Research Consortium, list available at www.gemproject.ca.

**Funding Agencies:**

CCC, CIHRHelmsley Charitable Trust

